# The Potential Impact of COVID-19 on Health-Related Quality of Life in Children and Adolescents: A Systematic Review

**DOI:** 10.3390/ijerph192214740

**Published:** 2022-11-09

**Authors:** Si Nae Ahn

**Affiliations:** Department of Occupational Therapy, Cheongju University, Cheongju 28503, Korea; otlovesn@cju.ac.kr

**Keywords:** adolescents, children, COVID-19, quality of life

## Abstract

This was a systematic review of studies examining the effect of COVID-19 on the health-related quality of life (HRQoL) of children and adolescents. The review was conducted by examining the current literature and analyzing up-to-date evidence. The studies were extracted from three major databases (CINAHL Complete, MEDLINE, and Web of Science) and analyzed. Studies on children and adolescents whose HRQoL has been affected by COVID-19 were included based on the eligibility criteria. Ultimately, eight studies met these criteria. The evidence of the selected studies was analyzed; the research design, age categories, respondents, evaluation tools, gender differences, and variability before and during COVID-19 were systematically reviewed. This review found differences in these groups regarding oral symptoms, functional limitations, emotional well-being, and social well-being. Furthermore, this review highlighted the relative paucity of studies that comprehensively investigate the latest evidence of changes in the HRQoL of children and adolescents due to COVID-19 in preparation for the post-COVID era.

## 1. Introduction

At the end of January 2020, the World Health Organization (WHO) classified the “Coronavirus Disease 2019 (COVID-19)” as a global health emergency [1]. Subsequently, the world entered a pandemic. Owing to COVID-19 lockdown measures, children and other young people have undergone various social changes, including lockdowns, school closures, contact restrictions, and recreational activity restrictions. The pandemic forced them to stay home to protect themselves from interpersonal contact. As such, careful evaluation of children and adolescents’ lifestyle and psychological changes due to the pandemic is necessary [2,3,4] to better understand possible undesirable consequences, mitigate potential negative effects, and smooth the transition back to normalcy [3]. Previous studies have suggested that these COVID-19 mitigation strategies have had profound mental and physical health consequences on students [5,6]. These include significant decreases in physical activity levels [7,8,9,10] and higher rates of mental health issues reported among children and adolescents during the pandemic [4,11]. Similarly, a recent meta-analysis found that mental health problems increased significantly during the pandemic [12]. Furthermore, previous studies have suggested that the COVID-19 pandemic may have had profound social, economic, and psychosocial consequences on children and adolescents, prompting further research [4,5,6,7,10,11,12].

The International Classification of Functioning, Disability, and Health (ICF), a standard for the classification of disability centered on living functions proposed by the WHO [13], was used in the present study. The ICF provides a scientific framework comprising questions on bodily functions, structures, activities, participation, and environmental factors. It covers key life-related functions at different stages of life. The international classification of functioning, disability, and health—children and youth version (ICF-CY) was created based on the ICF. The functional area, “activity and participation”, was used as a conceptual framework to measure education, health, and social life. These factors affect children’s environment and development [14]. According to the ICF-CY, activities and participation are integral to a child’s development. However, quality of life is also a critical factor requiring attention in the promotion of physical and mental health [15].

An individual’s subjective perception of their satisfaction with their health is directly related to the psychosocial state of the individual in question [16]. COVID-19 is closely related to children and adolescents’ physical and psychosocial development [4,5,6,7,8,9,10,11,12]. Strictly maintaining quarantine and social distancing rules requires substantial sacrifice, as these measures often conflict with fundamental aspects crucial to young people’s HRQoL. During the pandemic, feelings of loneliness and isolation likely increased among children and adolescents. Moreover, this viral outbreak caused uncertainty and anxiety among everyone, likely affecting the well-being of children and adolescents [3]. Although scholars have studied the changes in children and adolescents’ physical and psychosocial situations, studies related to their quality of life are still lacking.

Measuring the HRQoL of children and adolescents remains a relatively new area of research compared with adults’ HRQoL [17]. However, because of the COVID-19 outbreak, interest in the quality of life of children and adolescents is increasing. Children’s quality of life needs to be studied to understand their development, especially during the post-COVID-19 era. However, studies in this context are still lacking. A recent systematic review suggests that the pandemic negatively affected the general HRQoL of both children and adolescents [18]. However, evidence regarding gender differences and other specific HRQoL dimensions is lacking, with pre- and during-pandemic periods not adequately compared. Accordingly, up-to-date evidence and analyses are needed during this transition into the post-COVID era. Similarly, research is necessary due to the limited investigations on changes in children and adolescents’ quality of life before and during the pandemic. Therefore, physical and psychological HRQoL factors among younger people during the pandemic must be investigated.

Hence, this review investigated changes in child and adolescent HRQoL before and during the pandemic to prepare for the post-COVID era. This was achieved through a systematic literature review examining up-to-date comprehensive evidence and analyzing differences according to gender, respondents, and evaluation tools.

## 2. Materials and Methods

### 2.1. Study Design

**Figure 1:**
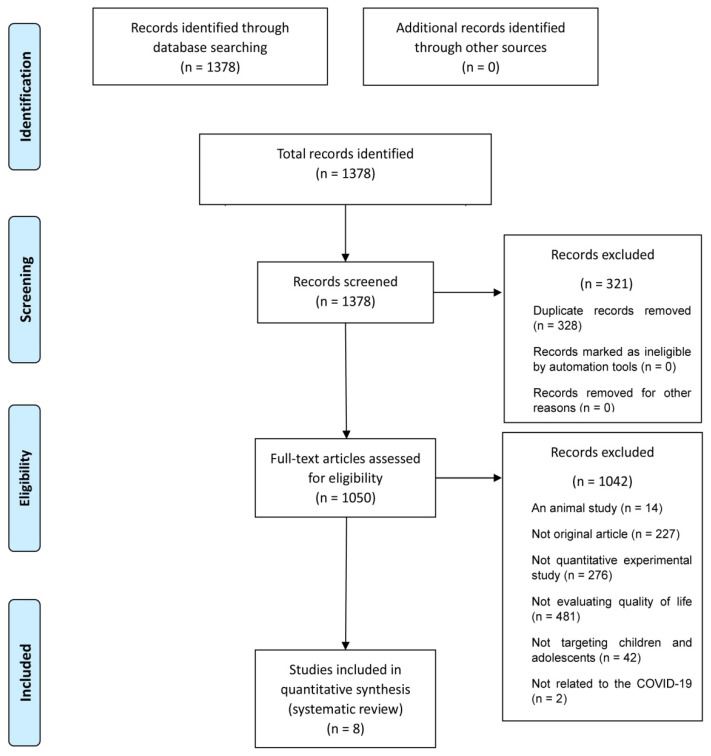
PRISMA flow diagram.

This study reviewed the HRQoL factors of children and adolescents affected due to COVID-19. A systematic literature review was conducted that followed the guidelines of the 2020 Preferred Reporting Items for Systematic Reviews and Meta-Analyses statement [19]. A flow diagram of this study’s process is shown in Figure 1.

### 2.2. Search Strategy

The study’s strategy involved searching for keywords in three scientific databases: CINAHL Complete (https://www.ebsco.com/products/research-databases/cinahl-complete, accessed on 31 March 2022), MEDLINE (https://www.nlm.nih.gov/medline, accessed on 31 March 2022), and Web of Science (www.webofscience.com, accessed on 31 March 2022). The following keywords were used: “coronavirus” and “child”, “coronavirus” and “child’s”, “coronavirus” and “children”, “coronavirus” and “children’s”, “coronavirus” and “adolescence”, “coronavirus” and “adolescences”, “coronavirus” and “adolescent.” The literature search criteria included published articles reviewed according to this study’s selection and exclusion criteria.

### 2.3. Study Selection Process

The inclusion criteria were studies written in English and available in full text, studies on children and adolescents, studies utilizing experimental quantitative designs, studies evaluating quality of life, and studies related to COVID-19. The exclusion criteria were research on animals, studies not targeting children and adolescents, studies that did not include an experimental quantitative design, and studies that did not include either quality of life or COVID-19. Furthermore, only original studies published in peer-reviewed journals were considered.

A total of 1378 records were identified after entering the chosen keywords into the database searches. Subsequently, the study titles were reviewed to remove any duplicates. Abstracts and full texts were reviewed based on our inclusion and exclusion criteria. Ultimately, eight studies met the eligibility criteria for inclusion in this study.

### 2.4. Risk of Bias

The Evidence Project tool was used to assess the risk of bias in this study. The tool was developed by the Evidence Project, which conducts systematic reviews and meta-analyses. The tool comprises eight items: cohort, control or comparison group, pre–post intervention data, random assignment of participants to the intervention, random selection of participants for assessment, follow-up rate of 80% or more, comparison group’s equivalent on sociodemographic factors, and comparison groups equivalent at baseline on outcome measures. This tool provides the following options for each item: “no”, “yes”, “not applicable” or “not reported” [20].

### 2.5. Data Extraction and Analysis

The collected data were extracted from the eligible studies. The following variables were extracted: author, year of publication, country, study design, population, respondents, gender differences, and outcome measurements related to HRQoL, and statistical data. Synthesis of results was provided with an initial description of the study population, including each study design and the total sample size and age range. The results of this review are derived by dividing age groups according to classification by stages of development [21]. The study also compiles a summary of the tools used to assess HRQoL and the impact of COVID-19 on the participating children and adolescents in each study. A summary with key findings was extracted for each study. Owing to the heterogeneity of the study designs and included articles, it was not possible to calculate estimated effects across studies with confidence intervals.

## 3. Results

### 3.1. Study Selection

A database search identified 1378 studies. After review, 321 duplicates were excluded. The remaining 1050 studies were assessed for eligibility upon review of their abstracts and full texts. A total of 1042 studies were excluded based on the following exclusion criteria: studies that did not include humans (*n* = 14), studies that were not original articles (*n* = 227), studies that did not use a quantitative experimental design (*n* = 276), studies that were not evaluated using a quality of life assessment tool (*n* = 481), studies whose participants were not the target population (*n* = 42), and studies that were not related to COVID-19 (*n* = 2). Ultimately, eight studies met the eligibility criteria and were included in the final data extraction. The study selection process is summarized in Figure 1.

### 3.2. Study Design

All studies selected for this systematic review involved a cross-sectional design.

### 3.3. Risk of Bias in the Included Studies

Bias risk was assessed using the Evidence Project risk of bias tool, which includes eight criteria. The eight studies selected for this study were evaluated to ensure they met one to three of these criteria. Of the eight criteria, two studies (25.0%) met one [22,23], three studies (37.5%) met two [24,25,26], and three studies (37.5%) met three (Table 1) [27,28,29].

### 3.4. Participant Characteristics

The samples from the eight studies comprised 20,509 participants, with an average of 2563.6 ± 4355.2 per study (207–13,002 participants).

### 3.5. The Respondents and Countries in the Included HRQoL Studies

Analysis of the studies on HRQoL by age group showed that four (50.0%) targeted early to late adolescents [22,23,25,29], three (37.5%) targeted respondents from middle childhood to early adolescence [24,26,27], and one (12.5%) targeted only respondents in middle childhood [28]. Analysis of respondents revealed that one study used responses from parents (12.5%), three studies collected responses from children and adolescents (37.5%), and four studies collected responses from adolescents only (50.0%) (Table 2).

By country, the United States had the highest number of studies, while the other studies were from Norway, Spain, southern Germany, and France. Studies in the United States and Spain compared the HRQoL of children and adolescents before and after COVID-19 quarantines [24,26]. Studies conducted in southern Germany and the United States compared the HRQoL of children and adolescents before and during the pandemic [25,28]. Studies comparing the HRQoL of children and adolescents at one time point during the pandemic were conducted in France, the United States, and Norway (Table 3) [22,23,27,29].

### 3.6. HRQoL Outcome Measurement Tools

The outcome measurements for examining the HRQoL of children and adolescents included the Pediatric Quality of Life Inventory (PedsQL), KIDSCREEN-10 or KIDSCREEN-27, KINDL-R, and the Child Perceptions Questionnaire for children aged 11 to 14 years (CPQ11-14).

The frequency analysis of these measurement tools for children and adolescents revealed that the distribution of measurements for evaluation was as follows. Three studies (37.5%) used the PedsQL [22,25,27,29], three studies (37.5%) used either KIDSCREEN-10 [23,26] or KIDSCREEN-27 [27], one study (12.5%) used KINDL-R [28], and one study (12.5%) used the CPQ11–14 [24] (Table 3).

### 3.7. HRQoL by Gender

When each study was compared according to their focus on gender, we found that five (62.5%) reported differences in HRQoL according to gender [23,25,26,27,28], while the remaining three (37.5%) did not [22,24,29]. For example, Kurz et al. reported that changes in HRQoL before and during the pandemic differed by gender [28]. Bourion-Bédès et al. compared the quality of life of children and adolescents by gender [27]. When they compared HRQoL scores by gender and age group (8–11 years old and 12–18 years old), they found that self-reported quality of life for physical well-being, psychological well-being, and school environment were significantly lower in the older girl group than in the other groups. McGuine et al. compared the quality of life of females and males during the pandemic and found that the former scored lower on HRQoL than the latter [25]. Two of the five studies that reported differences in HRQoL factors according to gender presented statistical analysis results. Riiser et al. found a statistically [23] positive correlation between HRQoL and gender, with boys being positively associated with HRQoL to a statistically significant degree (95% CI [4.5:8.3]). Furthermore, Vallejo Slocker et al. [26] reported gender differences in their participants’ HRQoL; the T-score of the KIDSCREEN-10 index indicated that males (*n* = 236, M = 51.04, SD = 9.89) scored higher than females (*n* = 223, M = 48.89, SD = 10.01), indicating better functioning. There were also gender differences in the HRQoL of the post-outbreak group, with males reporting lower HRQoL scores [26] (Table 2).

### 3.8. Differences in HRQoL before and during the Pandemic

Four studies compared HRQoL before and during the pandemic [22,24,26,28]. In McGuine et al. [22], the HRQoL of a pre- and during-COVID-19 athlete group was compared. Athletes in the pre-COVID-19 group reported higher overall HRQoL scores than those in the during-COVID-19 group. However, Vallejo Slocker et al. [26] reported no statistically significant difference between pre- and during-COVID-19 groups when comparing HRQoL. In addition, Knorst et al. evaluated the impact of the COVID-19 pandemic on oral HRQoL among adolescents [24]. According to their results, the negative effects of oral symptoms on the quality of life of adolescents during the pandemic period were low.

The remaining four studies analyzed HRQoL during the pandemic [23,25,27,29]. For example, Bourion-Bédès et al. compared the quality of life of children and adolescents during the pandemic and reported that adolescents had lower HRQoL scores [27]. McGuine et al. also compared the HRQoL of adolescents who played sports during the pandemic with those who did not and reported that the former had higher HRQoL scores [29].

## 4. Discussion

In light of the current transition to a post-COVID era, this study examined the latest evidence and analyses on changes in the quality of life of children and adolescents due to COVID-19 through a systematic review. Eight cross-sectional studies were selected according to the inclusion and exclusion criteria, comprising 20,509 participants. Studies reporting on the quality of life of children and adolescents in the context of COVID-19 were obtained from the United States, Norway, Spain, southern Germany, and France. Studies from the United States and Spain compared the HRQoL of children and adolescents before and after quarantine due to COVID-19 [24,26]. Studies from southern Germany and others from the United States compared the HRQoL of children and adolescents before and during the pandemic [25,28]. The remaining studies from France, the United States, and Norway compared the HRQoL of children and adolescents at one time point during the pandemic [22,23,27,29]. The results of these studies indicate little significance between gender and HRQoL during the pandemic. Regarding differences in HRQoL pre- and post-pandemic, some studies identified pathologically significant differences in oral symptoms, functional limitations, emotional well-being, and social well-being. Others found significant differences in physical and psychosocial health pre- and post-pandemic. Additionally, certain studies looked at the difference in HRQoL among people who played sports and those who did not. They found that people who did not play a sport had a lower psychosocial score, which was statically significant.

Kurtz et al. [28] compared the impact of COVID-19 on children by gender. They found different results for males and females, with the latter reporting that their mental health was significantly more affected. McGuine et al. [25] reported on the HRQoL of adolescents and found that girls were less physically active, had lower HRQoL scores, and had higher depressive symptoms than their male counterparts. However, this gender difference was not verified in terms of any causal relationship. Hence, it cannot be said that restrictions on school attendance and participation in activities following social distancing or lockdown measures due to COVID-19 had any direct impact. Gender differences may have played a role in the HRQoL of children and adolescents during the COVID-19 pandemic. However, this would have been combined with other factors, such as personal challenges, lack of social interaction, economic uncertainty, and access to social media [6,30,31].

In this review, we found low correlation between HRQoL and gender during the COVID-19 pandemic. This review included studies on comparisons with the post-COVID-19 period. This is meaningful in that we covered aspects of HRQoL that are important to children and adolescents’ development and social engagement during COVID-19. Previous studies have shown that the HRQoL of young children is negatively affected by infectious diseases, more so when compared to older children [27]. However, among the studies included in this review, current HRQoL research includes more adolescents than children. Psychological distress in children can affect their HRQoL and negatively affect their long-term development, particularly for those who face social restrictions and limited social interaction. Therefore, it is necessary to investigate the HRQoL of younger children further.

Despite these findings, the HRQoL of children and adolescents remains relatively unexplored compared to adults [17]. The quality of life and background of younger people who have experienced changes due to the COVID-19 outbreak should be considered to understand their development in the post-COVID era. Thus, this study is meaningful as it provides insight into the HRQoL of children and adolescents.

This systematic review reflects the recent growing interest in the HRQoL of children and adolescents. Although this review provides evidence of changes in the HRQoL of children and adolescents due to COVID-19, more work is needed to demonstrate the impact of factors such as personal background, gender differences, and different respondents. This systematic review has the following limitations. First, the reviewed literature included only a few published studies. Second, a limited number of databases were searched. Third, our search keywords were similarly limited. Fourth, detailed descriptions were lacking for direct comparisons in each study (general characteristics of the selected papers are provided in Table 2 for comparison). Fifth, most selected studies have inadequate evidence and did not use probabilistic sampling methods. Therefore, there is a limit to the generalizability of their results. Future research should be conducted using more extensive search terms and keywords while also including HRQoL studies across numerous databases in order to compensate for these limitations.

## 5. Conclusions

This systematic review investigated changes in HRQoL caused by COVID-19 among children and adolescents to prepare for the post-COVID era. The study comprehensively investigates the latest evidence on this topic through analyses of differences in gender and evaluation tools. These findings summarize the general characteristics of the HRQoL of children and adolescents and provide a basis for future studies and interventions. Furthermore, this systematic review confirms the importance of changes in HRQoL regarding oral symptoms, functional limitations, emotional well-being, and social well-being of children and adolescents due to the COVID-19 pandemic. Furthermore, this review highlights the relative paucity of studies that comprehensively investigate the latest evidence in a post-COVID era. This must be accomplished by examining changes in the HRQoL of children and adolescents due to COVID-19 to prepare for the post-COVID era. Lastly, there is a need to verify the objective validity of how changes in the HRQoL of children and adolescents who have experienced COVID-19 affect their development from a long-term perspective. Hence, more research on this subject is needed.

## Figures and Tables

**Table 1:** Risk of bias in included articles assessed using the Evidence Project risk of bias tool (*n* = 8).

Authors	Cohort	Control or Comparison Group	Pre/Post Intervention Data	Random Assignment of Participants to the Intervention	Random Selection of Participants for Assessment	Follow-Up Rate of 80% or More	Comparison Groups Equivalent on Sociodemographic Factors	Comparison Groups Equivalent at Baseline on Disclosure
Kurz et al. [28]	Yes	Yes	Yes	NA	NA	NA	NA	NA
Bourion-Bédès et al. [27]	Yes	Yes	No	No	No	Yes	NR	NA
Vallejo Slocker et al. [26]	Yes	No	Yes	NA	NA	NA	NA	NA
Knorst et al. [24]	Yes	No	Yes	NA	NA	NA	NA	NA
McGuine et al. [25]	Yes	No	Yes	NA	NA	NA	NA	NA
McGuine et al. [22]	Yes	No	No	NA	NA	NA	NA	NA
McGuine et al. [29]	Yes	Yes	No	NA	NA	NA	Yes	NR
Riiser et al. [23]	Yes	No	No	NA	NA	NA	NA	NA

NA: not applicable; NR: not reported.

**Table 2:** Results of the respondent types in the included HRQoL studies (*n* = 8).

Authors	Age	Age Group	Respondent of HRQoL	Presence or Absence of Gender Differences
Kurz et al. [28]	6 to 7 years	Middle childhood	Parents	Present
Bourion-Bédès et al. [27]	8 to 18 years	Middle childhood, early adolescence	Children and adolescents	Present
Vallejo Slocker et al. [26]	8 to 18 years	Middle childhood, early adolescence	Children and adolescents	Present ^a^
Knorst et al. [24]	10 to 15 years	Middle childhood, early adolescence	Children and adolescents	Absent
McGuine et al. [25]	13 to 19 years	Early adolescence, late adolescence	Adolescents	Absent
McGuine et al. [22]	13 to 19 years	Early adolescence, late adolescence	Adolescents	Present
McGuine et al. [29]	13 to 19 years	Early adolescence, late adolescence	Adolescents	Absent
Riiser et al. [23]	16 to 19 years	Early adolescence, late adolescence	Adolescents	Present ^a^

^a^ A statistical analysis of gender differences was conducted.

**Table 3:** Main findings of the HRQoL cross-sectional studies of children and adolescents during the COVID-19 pandemic (*n* = 8).

Authors	Country	Population Sample Size (*n*)	HRQoL			
			Outcome Measure	Pre-Pandemic ^a^	During-Pandemic ^a^	*p*-Value
Bourion-Bédès et al. [27]	France	Children and adolescents (*n* = 471)	KIDSCREEN-27		*8–11-year-old girls/boys* Physical well-being 49.0 (9.6)/50.0 (9.9) Psychological well-being 51.5 (9.9)/50.9 (8.5) Parent and autonomy 46.2 (10.4)/46.5 (8.8) Social support and peers 30.6 (14.2)/32.2 (17) School 50.5 (10.1)/49.4 (9.6) *12–18-year-old girls/boys* Physical well-being 42.1 (9.9)/45.4 (10) Psychological well-being 44.7 (9.5)/50.3 (10.1) Parent and autonomy 48.4 (12.6)/48.9 (11.7) Social support and peers 41.8 (13.2)/37.1 (12.6) School 47.4 (10.3)/46.4 (10.2)	Physical well-being <0.0001 Psychological well-being <0.0001 Parent and autonomy 0.19 Social support and peers <0.0001 School 0.015
Knorst et al. [24]	United States	Children and adolescents (*n* = 207)	CPQ11-14 (oral HRQoL)	*Before COVID-19* Oral symptoms 3.7 (2.5) Functional limitations 2.7 (2.8) Emotional well-being 2.6 (3.3) Social well-being 1.6 (2.3) Total score 10.8 (8.1)	*During COVID-19* Oral symptoms 3.2 (2.5) Functional limitations 2.0 (2.7) Emotional well-being 1.5 (2.8) Social well-being 0.9 (1.9) Total score 7.7 (7.5)	Oral symptoms <0.01 Functional limitations <0.01 Emotional well-being <0.01 Social well-being <0.01 Total score <0.01
Kurz et al. [28]	Southern Germany	Children (*n* = 362)	KINDL-R	First HRQoL girls/boys 79.5 (9.1)/73.6 (11.7) Second HRQoL girls/boys 85.1 (8.1)/82.8 (6.4) Third HRQoL girls/boys 86.3 (7.1)/83.6 (10.5)	Girls/boys 80.8 (8.6)/82.1 (9.0)	Girls/boys 0.002/0.06
McGuine et al. [25]	United States	Adolescent athletes (*n* = 3243)	PedsQL	Physical summary 91.7 (91.3, 92.1) ^b^ Psychosocial summary 90.4 (89.9, 90.8) ^b^ Total 90.9 (90.5, 91.3) ^b^	Physical summary 82.6 (82.2, 83.0) ^b^ Psychosocial summary 76.2 (75.8, 76.6) ^b^ Total 78.4 (78.0, 78.8) ^b^	<0.001 <0.001 <0.001
McGuine et al. [22]	United States	Adolescent athletes (*n* = 13,002)	PedsQL		*Total* Physical summary 80.3 (79.5, 81.0) ^b^ Psychosocial summary 74.8 (74.0, 75.6) ^b^ Total 76.7 (76.0, 77.5) ^b^ *Female* Physical summary 77.9 (77.1, 78.6) ^b^ Psychosocial summary 72.9 (72.0, 73.7) ^b^ Total 74.6 (73.8, 75.4) ^b^ *Male* Physical summary 83.8 (82.9, 84.6) ^b^ Psychosocial summary 77.7 (76.8, 78.6) ^b^ Total 79.8 (79.0, 80.6) ^b^	None
McGuine et al. [29]	United States	High school athletes (*n* = 559)	PedsQL		*Played a fall sport* Physical summary 92.3 (90.1, 94.4) ^b^ Psychosocial summary 86.4 (83.3, 89.4) ^b^ Total 88.4 (85.9, 90.9) ^b^ *Did not play a fall sport* Physical summary 86.5 (84.1, 88.9) ^b^ Psychosocial summary 75.9 (72.5, 79.3) ^b^ Total 79.6 (76.8, 82.4) ^b^	Physical summary 0.004 Psychosocial summary <0.001 Total <0.001
Riiser et al. [23]	Norway	Adolescents (*n* = 2206)	KIDSCREEN-10		55.3 (17.0) *Girls* None *Boys* (4.5, 8.3) ^b^	
Vallejo Slocker et al. [26]	Spain	Children and adolescents (*n* = 459)	KIDSCREEN-10	*Before COVID-19* 50.4 (10.1)	*After COVID-19* 50 (10.0) *Gender* Males 2.9 (2.17) Females 3.8 (2.46)	Outbreak >0.4 Gender 0.021

^a^ M(SD); ^b^ 95% CI; CPQ11–14: Child Perceptions Questionnaire for children aged 11 to 14 years; df: degrees of freedom; HRQoL: health-related quality of life; JSLE: juvenile systemic lupus erythematosus; JIA: juvenile idiopathic arthritis; PedsQL: Pediatric Quality of Life Inventory.

## Data Availability

The systematic review data used to support the findings of this study are included within the article.
